# Occult Bacteraemia and Aortic Graft Infection: A Wolf in Sheep's Clothing

**DOI:** 10.1155/2013/968542

**Published:** 2013-12-29

**Authors:** E. Trautt, S. Thomas, J. Ghosh, P. Newton, A. Cockcroft

**Affiliations:** University Hospital of South Manchester, Manchester, UK

## Abstract

We report a case of late-onset aortic prosthetic vascular graft infection. We stress the importance of maintaining a high index of suspicion for any patient presenting with fever on the background of in situ prosthetic material. We present the difficulties in managing these extremely complicated, often life and limb threatening infections and suggest that a multidisciplinary team approach, involving specialist centre referral, may be key to success. We highlight the difficulties in diagnosing late-onset PVGI, where presentation can be subacute with subtle signs and confusing microbiology. In this case the presentation was pyrexia of unknown origin with multiple positive blood cultures isolating a variety of gut-associated organisms; *a wolf in sheep's clothing*.

## 1. Background

Prosthetic vascular graft infection (PVGI) is a significant complication of arterial reconstructive surgery [1]. The incidence has been reported to vary from 1 percent to 6 percent [2], depending on the site of the graft (infrainguinal 2–5%, aortofemoral 1-2%, and aortic 1%). Although the relative risk of PVGI is low, the clinical consequences of an infected vascular graft can be catastrophic for the patient, with an associated operative morbidity of 40–70% (limb amputation rates of up to 70% for lower extremity grafts [3]) and a recognized mortality rate of 30–50% (up to 75% with intra-abdominal aortic grafts [4]).

We describe an unusual presentation of late-onset aortic graft infection which stresses the importance of maintaining a high index of suspicion in any patient with unexplained fevers and underlying in situ prosthetic material. In management of this case we demonstrate effective clinical use of Daptomycin and highlight the need for national consensus guidelines to guide the management of these complex infections.

## 2. Case Report

This 79-years-old gentleman was admitted from the Infectious Diseases clinic, in April 2011. He presented to the clinic with night sweats, intermittent fevers, rigors, lethargy, weight loss, poor appetite, and generalised arthralgias. He described a change in the bowel habit over the previous few months with constipation and mild abdominal discomfort. His C-reactive protein was 58. This was his fourth follow-up clinic appointment following a recent hospital discharge.

His past medical history included the following: August 2010 admission for relapsed septic arthritis of a right native knee, joint fluid aspirated at that time isolated *Pseudomonas aeruginosa* and blood cultures repeatedly isolated *Pseudomonas aeruginosa* and *Enterobacter*; July 2010 admission for probable recurrent septic arthritis, blood cultures isolated *Streptococcus constellatus* and *Aerococcus*; December 2009 admission for a primary septic arthritis, culture of synovial fluid isolated *Streptococcus constellatus*. In 1993 he had undergone an aortic aneurysm repair with insertion of an aorto-bi-iliac Dacron surgical graft.

On his admission in December 2009, because of the in situ aortic graft, he had undergone a CT abdomen and pelvis which showed normal appearances of the aorto-bi-iliac graft. The scan was repeated on each subsequent admission, in July 2010 and August 2010, each time showing normal appearances of the graft and no evidence of a fluid or gas collection around the abdominal aorta or iliac arteries. On admission from clinic, he underwent investigation for PUO in which he had multiple sets of blood cultures collected and 3 sets of isolated lactobacillus (Table 1).

He underwent a whole body scan showing a pool of activity in the right knee suggestive of chronic low-grade persistent infection. A transthoracic echocardiogram and a colonoscopy to investigate the altered bowel habit were carried out and were both negative. At this time a fourth repeat CT abdomen and pelvis was requested. This showed small extramural pockets of gas at the level of the graft bifurcation and a further possible tiny pocket of gas at the cranial aspect of the graft, appearances which would be suspicious for sepsis related to the graft.

At this point, there was multidisciplinary team input from vascular surgeons, vascular radiologists, infectious diseases and microbiology and appropriate management options were discussed. The two available options were (1) explantation of the graft with extra-anatomical bypass and (2) long-term suppressive antibiotics with serial CRP and imaging. In view of his age, that the WCC was within normal limits and the CRP was falling, the equivalence of the CT finding, and the fact that the surgery to remove the graft would carry a significant mortality and morbidity risk, the decision was made to start IV antibiotics for a minimum of 6 wks. The caveat to this approach was that in the event of worsening sepsis despite antibiotics and/or CT evidence of worsening perigraft infection or development of aortoenteric fistula, then surgery would be carried out.

IV Daptomycin (6 mg/kg) (plus gentamicin initially) was commenced after confirmatory MIC testing of the lactobacillus to Daptomycin; this was the most recently and most persistently isolated pathogen. Daptomycin was well tolerated by the patient. At 6 weeks, based on good clinical response, this was changed to oral amoxicillin plus clindamycin, and the patient was discharged home. Within 48 hrs of discharge he represented with rigors and fevers. He was readmitted and a repeat CT showed increasing air encircling the right most anterior limb of the aortic graft. At this point the decision was made to carry out surgery to remove the graft.

**Figure 1:**
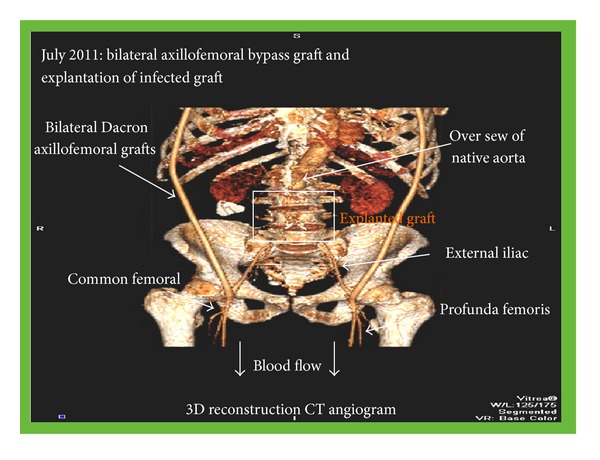
3D-CT angiogram demonstrating removal of the infected aortic graft and extra-anatomical bypass.

In July 2011, the infected graft was explanted, the space washed out, and bilateral axillofemoral bypass graft inserted. Intraoperatively, a perforated duodenum was reported, which was the likely source of the multiple bacteraemias isolating the variety of gut organisms seen. This was repaired involving a retrocolic gastrojejunostomy and feeding jejunostomy, in an operation lasting more than 10 hours (Figure 1).

To date, the patient remains well at home, the graft remains patent and perfusing the leg, and he has chosen not to continue oral suppressive antibiotics.

## 3. Discussion

Diagnosis of PVGI is extremely challenging. It is based on patient history and examination and parameters such as CRP, WCC, blood cultures (which often remain negative in late-onset graft infection), and 8 radiological imaging, which can be extremely difficult to interpret and in this case was repeatedly negative in the early stages. Unlike early-onset PVGI, which is often attributed to contamination of the graft at or soon after insertion [1] and presents mainly as a postsurgical wound infection, the etiology of late-onset graft infections is far less certain. Presentation can be more occult in manner and many patients present with few or no overt symptoms of disease [1]. There may be local signs of infection such as thrombosis or failure of the graft, false aneurysm, or bleeding (aortoenteric fistula formation). In this case the patient presented with occult signs of graft-duodenal erosion, resulting in the translocation of a variety of gut organisms into the blood stream, likely seeding a potentially osteoarthritic knee joint, which was the initial presenting complaint.

Gold standard treatment involves complex surgery to remove the infected graft. For many patients, however, multiple comorbidities often render this an unfeasible option, leaving long courses of often empirical broad spectrum intravenous antibiotics as the only alternative. A lack of data on the effectiveness of some of the newer antimicrobial agents in prosthetic graft infection limits the treatment options available. In this case Daptomycin was used for the suppression of infection. This was an off-licence use of this newer class of antimicrobial agent, and the data to support its use is lacking. Despite the fact that management will often be on a patient-by-patient basis, there is a real and urgent need to develop national consensus treatment guidelines, providing advice on the use of newer antimicrobial agents such as Daptomycin, Linezolid, and Tigecycline in PVGI and on the optimal duration of therapy.

## Figures and Tables

**Table 1:** Microbiology results.

Date	Clinical situation	Microbiology		Imaging
		Sample site	Culture results	
Dec 2009	Inpatient admission Septic arthritis	Synovial fluid	*S. constellatus* (enrichment only)	Jan. 2010 CT abdomen with contrast: no acute abnormality detected
Apr 2010	Inpatient admission Relapsed septic arthritis	Blood	*S. constellatus* *Aerococcus urinae *	Jul. 2010 CT abdomen and pelvis: no evidence of graft infection
Aug 2010	Inpatient admission Recurrent septic arthritis	Joint fluid Blood (x3)	*Pseudomonas aeruginosa Pseudomonas aeruginosa Enterobacter cloacae *	Aug. 2010 CT angiogram aorta: no evidence of abdominal aortic stent graft infection
Mar 2011	Outpatient clinic	Blood	*Lactobacillus* species	
Apr 2011	Outpatient clinic Admitted with PUO	Blood (x4)	*Lactobacillus paracasei* *Lactobacillus* species *S. oralis *	Apr. 2011 CT abdomen and pelvis: at least 2 small droplets of retroperitoneal gas which appear extraluminal and suspicious for sepsis related to the graft
